# Supplementary material for: HIV-1 integrase resistance associated mutations and the use of dolutegravir in Sub-Saharan Africa: A systematic review and meta-analysis
Source: PLOS Glob Public Health. 2022 Oct 11;2(10):e0000826. doi: 10.1371/journal.pgph.0000826 (PMC10021461; doi:10.1371/journal.pgph.0000826)
Supplement: S1 Appendix — (DOCX) [file pgph.0000826.s002.docx]

**S1 Appendix:** List of papers excluded and reasons for exclusion

| First author | Year of publication | Title of the paper | Reasons of Exclusion |
| --- | --- | --- | --- |
| Isabelle Malet | 2008 | Mutations associated with failure of raltegravir treatment affect integrase sensitivity to the inhibitor in vitro | Patients not living in SSA |
| Almoustapha-Issiaka Maïga | 2009 | Genetic barriers for integrase inhibitor drug resistance in HIV type-1 B and CRF02_AG subtypes. | Patients/participants are INSTI-unexperienced |
| Isabelle Malet | 2009 | Quasispecies variant dynamics during emergence of resistance to raltegravir in HIV-1-infected patients | Patients not living in SSA |
| Sebastiaan J van Hal | 2009 | HIV-1 integrase polymorphisms are associated with prior antiretroviral drug exposure | Patients/participants are INSTI-unexperienced |
| Anne Depatureaux | 2010 | Specific diagnosis and follow-up of HIV-1 group O infection: RES-O data | Patients not living in SSA |
| Francesca Ceccherini-Silberstein | 2010 | Secondary integrase resistance mutations found in HIV-1 minority quasispecies in integrase therapy-naive patients have little or no effect on susceptibility to integrase inhibitors | Patients/participants are INSTI-unexperienced |
| Maria A. Papathanasopoulos | 2010 | Characterization of HIV type 1 genetic diversity among South African participants enrolled in the AIDS Vaccine Integrated Project (AVIP) Study | Patients/participants are INSTI-unexperienced |
| Muhammad Q. Fish | 2010 | Natural Polymorphisms of integrase among HIV Type 1-Infected South African Patients | Patients/participants are INSTI-unexperienced |
| Annette H. Sohn | 2010 | Sequencing of antiretroviral therapy in children in low-and middle-income countries | This is a review or systematic review |
| Damian J. McColl | 2010 | Strand transfer inhibitors of HIV-1 integrase: Bringing IN a new era of antiretroviral therapy | This is a review or systematic review |
| Carolina Garrido | 2010 | Integrase variability and susceptibility to HIV integrase inhibitors: impact of subtypes, antiretroviral experience and duration of HIV infection | Patients not living in SSA |
| Nazle Mendonca Collaço Véras | 2011 | Molecular Epidemiology of HIV Type 1 CRF02_AG in Cameroon and African Patients Living in Italy | Patients/participants are INSTI-unexperienced |
| Geoffrey S. Gottlieb | 2011 | Hiv-2 integrase variation in integrase inhibitor-naïve adults in senegal, west africa | Patients/participants are INSTI-unexperienced |
| Sarita D. Boyd | 2011 | Transmitted raltegravir resistance in an HIV-1 CRF-AG-infected patient | Patients not living in SSA |
| Ruth M Bland | 2011 | Management of HIV-infected children in Africa: Progress and challenges | This is a review or systematic review |
| Isabelle Malet | 2011 | Prevalence of resistance mutations related to integrase inhibitor S/GSK1349572 in HIV-1 subtype B raltegravir-naive and -treated patients | Patients not living in SSA |
| Ricardo Jorge Camacho | 2012 | Special aspects of the treatment of HIV-2-infected patients | This is a review or systematic review |
| Guinevere Q. Lee | 2012 | Prolonged and Substantial Discordance in Prevalence of Raltegravir-Resistant HIV-1 in Plasma versus PBMC Samples Revealed by 454 "Deep" Sequencing | Patients not living in SSA |
| Ombretta Turriziani | 2012 | Short Communication: Analysis of the Integrase Gene from HIV Type 1-Positive Patients Living in a Rural Area of West Cameroon | Patients/participants are INSTI-unexperienced |
| Michelli F. Oliveira | 2012 | Genetic Diversity and Naturally Polymorphisms in HIV Type 1 Integrase Isolates from Maputo, Mozambique: Implications for Integrase Inhibitors | Patients/participants are INSTI-unexperienced |
| Anthony Kebira Nyamache | 2012 | HIV Type 1 Genetic Diversity and Naturally Occurring Polymorphisms in HIV Type 1 Kenyan Isolates: Implications for Integrase Inhibitors | Patients/participants are INSTI-unexperienced |
| Agozie Ubesie C. | 2012 | Pediatric HIV/AIDS in sub-Saharan Africa: emerging issues and way forward | This is a review or systematic review |
| Marjorie Monleau | 2012 | Drug resistance mutations of HIV-1 non-B viruses to integrase inhibitors in treatment-naïve patients from sub-Saharan countries and discordant interpretations | Patients/participants are INSTI-unexperienced |
| Francesco Saladini | 2012 | Prevalence of HIV-1 integrase mutations related to resistance to dolutegravir in raltegravir naïve and pretreated patients | Patients not living in SSA |
| Daniele Armenia | 2012 | Study of genotypic and phenotypic HIV-1 dynamics of integrase mutations during raltegravir treatment: A refined analysis by ultra-deep 454 pyrosequencing | Patients not living in SSA |
| Isabelle Malet | 2012 | Risk factors for raltegravir resistance development in clinical practice | Patients not living in SSA |
| Amedeo Capetti | 2012 | 96 Week Follow-Up of HIV-Infected Patients in Rescue with Raltegravir Plus Optimized Backbone Regimens: A Multicentre Italian Experience | Patients not living in SSA |
| Brianna L. Kirk | 2013 | Early outcomes of darunavir-and/or raltegravir-based antiretroviral therapy in children with multidrug-resistant HIV at a pediatric center in Botswana | Full text unclear or unavailable |
| Joseph J. Eron | 2013 | Safety and Ef fi cacy of Dolutegravir in Treatment-Experienced Subjects With Raltegravir-Resistant HIV Type 1 Infection : 24-Week Results of the VIKING Study | Patients not living in SSA |
| Cindy Vavro | 2013 | Prevalent Polymorphisms in Wild-Type HIV-1 Integrase Are Unlikely To Engender Drug Resistance to Dolutegravir (S/GSK1349572) | Patients/participants are INSTI-unexperienced |
| Serge Clotaire Billong | 2013 | Population- based Monitoring of Emerging HIV-1 Drug B Resistance on Antiretroviral Therapy and Associated Factors in a Sentinel Site in Cameroon: Low Levels of Resistance but Poor Programmatic Performance Serge | Patients/participants are INSTI-unexperienced |
| Margaret L. McNairy | 2013 | Retention of HIV-infected children on antiretroviral treatment in HIV care and treatment programs in Kenya, Mozambique, Rwanda and Tanzania | Data Unclear (Authors weren't conntacted) |
| Mark Boyd | 2013 | Ritonavir-boosted lopinavir plus nucleoside or nucleotide reverse transcriptase inhibitors versus ritonavir-boosted lopinavir plus raltegravir for treatment of HIV-1 infection in adults with virological failure of a standard first-line ART regimen (SECOND-LINE): a randomised, open-label, non-inferiority study | Patients not living in SSA |
| Alessandra Fantauzzi | 2013 | Potential benefit of dolutegravir once daily: Efficacy and safety | This is a review or systematic review |
| Pascal Obong Bessong | 2013 | Genetic analysis of HIV-1 integrase sequences from treatment naive individuals in northeastern South Africa | Patients/participants are INSTI-unexperienced |
| Sharon L. Walmsley | 2013 | Dolutegravir plus Abacavir–Lamivudine for the Treatment of HIV-1 Infection | Patients not living in SSA |
| Anne-Geneviève Marcelin | 2013 | A cohort study of treatment-experienced HIV-1-infected patients treated with raltegravir: factors associated with virological response and mutations selected at failure | Patients not living in SSA |
| Elena Capel | 2013 | Significant changes in integrase-associated HIV-1 replication capacity between early and late isolates | Title/Abstract irrelevant |
| Antonella Castagna | 2014 | Dolutegravir in Antiretroviral-Experienced Patients With Raltegravir- and / or Elvitegravir- Resistant HIV-1 : 24-Week Results of the Phase III VIKING-3 Study | Patients not living in SSA |
| Mark Killick | 2014 | Generation and Characterization of an HIV-1 Subtype C Transmitted and Early Founder Virus Consensus Sequence | Title/Abstract irrelevant |
| Andreas Carganico | 2014 | New dolutegravir resistance pattern identified in a patient failing antiretroviral therapy | Full text unclear or unavailable |
| Andargachew Mulu | 2015 | Lack of integrase inhibitors associated resistance mutations among HIV-1C isolates | Patients/participants are INSTI-unexperienced |
| Mesplede Thibault | 2015 | The R263K substitution in HIV-1 subtype C is more deleterious for integrase enzymatic function and viral replication than in subtype B | Study did not involve participants |
| Fourati Slim | 2015 | Cross-resistance to elvitegravir and dolutegravir in 502 patients failing on raltegravir : a French national study of raltegravir-experienced HIV-1-infected patients | Patients not living in SSA |
| Santiago Moreno | 2015 | Eficacia de dolutegravir en pacientes experimentados: estudios SAILING y VIKING | The full text isn't writen in english or french |
| Peter K. Quashie | 2015 | Differential Effects of the G118R, H51Y, and E138K Resistance Substitutions in Different Subtypes of HIV Integrase | Study did not involve participants |
| Meintjes Graeme | 2015 | Third-line antiretroviral therapy in Africa: Effectiveness in a Southern African retrospective cohort study | Full text unclear or unavailable |
| Tomas Doyle | 2015 | Integrase inhibitor (INI) genotypic resistance in treatment-naive and raltegravir-experienced patients infected with diverse HIV-1 clades | Patients not living in SSA |
| Thibault Mesplède | 2015 | Resistance against integrase strand transfer inhibitors and relevance to HIV persistence | This is a review or systematic review |
| Mark A Boyd | 2015 | Baseline HIV-1 resistance, virological outcomes, and emergent resistance in the SECOND-LINE trial: An exploratory analysis | Patients not living in SSA |
| Joseph Fokam | 2015 | Monitoring HIV Drug Resistance Early Warning Indicators in Cameroon: A Study Following the Revised World Health Organization Recommendations | Study did not involve participants |
| Soo-Yon Rhee | 2015 | HIV-1 Drug Resistance Mutations: Potential Applications for Point-of-Care Genotypic Resistance Testing | Patients/participants are INSTI-unexperienced |
| Takahiro Seki | 2015 | Effects of Raltegravir or Elvitegravir Resistance Signature Mutations on the Barrier to Dolutegravir Resistance In Vitro | Study did not involve participants |
| Christopher E. Kandel | 2015 | Dolutegravir – A review of the pharmacology, efficacy, and safety in the treatment of HIV | This is a review or systematic review |
| Jiaming Liang | 2015 | The Combination of the R263K and T66I Resistance Substitutions in HIV-1 Integrase Is Incompatible with High-Level Viral Replication and the Development of High-Level Drug Resistance | Study did not involve participants |
| Said A. Hassounah | 2015 | Characterization of the Drug Resistance Profiles of Integrase Strand Transfer Inhibitors in Simian Immunodeficiency Virus SIVmac239 | Title/Abstract irrelevant |
| Jaqueline de Souza Cavalcanti | 2015 | High frequency of dolutegravir resistance in patients failing a raltegravir-containing salvage regimen | Patients not living in SSA |
| M. Casadella | 2015 | Primary resistance to integrase strand-transfer inhibitors in Europe | Title/Abstract irrelevant |
| Atsuko Hachiya | 2015 | Natural polymorphism S119R of HIV-1 integrase enhances primary INSTI resistance | Study did not involve participants |
| Salou Monerou | 2016 | High rates of virological failure and drug resistance in perinatally HIV-1-infected children and adolescents receiving lifelong antiretroviral therapy in routine clinics in Togo | Patients/participants are INSTI-unexperienced |
| Seth Inzaule | 2016 | Affordable HIV drug-resistance testing for monitoring of antiretroviral therapy in sub-Saharan Africa | Title/Abstract irrelevant |
| Alberto M. La Rosa | 2016 | Raltegravir in second-line antiretroviral therapy in resource-limited settings (SELECT): a randomised, phase 3, non-inferiority study | Patients not living in SSA |
| Sonia T. Boender | 2016 | Accumulation of HIV-1 drug resistance after continued virological failure on first-line ART in adults and children in sub-Saharan Africa | Patients/participants are INSTI-unexperienced |
| Aastha Gupta | 2016 | Projected uptake of new antiretroviral (ARV) medicines in adults in low- and middle-income countries: A forecast analysis 2015-2025 | Study did not involve participants |
| Mark A. Wainberg | 2016 | Might dolutegravir be part of a functional cure for HIV? | This is a review or systematic review |
| Colin M. Venner | 2016 | Infecting HIV-1 Subtype Predicts Disease Progression in Women of Sub-Saharan Africa | Title/Abstract irrelevant |
| Steve Kanters | 2016 | Comparative efficacy and safety of first-line antiretroviral therapy for the treatment of HIV infection: a systematic review and network meta-analysis | This is a review or systematic review |
| Andrew M Hill | 2016 | How can we achieve universal access to low-cost treatment for HIV? | This is a review or systematic review |
| Sonia T. Boender | 2016 | Protease Inhibitor Resistance in the First 3 Years of Second-Line Antiretroviral Therapy for HIV-1 in Sub-Saharan Africa | Patients/participants are INSTI-unexperienced |
| Claudia Hawkins | 2016 | HIV virological failure and drug resistance in a cohort of Tanzanian HIV-infected adults | Patients/participants are INSTI-unexperienced |
| Maureen Oliveira | 2016 | The M184I/V and K65R nucleoside resistance mutations in HIV-1 prevent the emergence of resistance mutations against dolutegravir | Study did not involve participants |
| Cissy Kityo | 2016 | HIV Drug Resistance Among Children Initiating First-Line Antiretroviral Treatment in Uganda | Patients/participants are INSTI-unexperienced |
| Ragna S. Boerma | 2016 | Suboptimal viral suppression rates among HIV-infected children in low- and middle-income countries: a meta-analysis | This is a review or systematic review |
| Raph L Hamers | 2016 | Enhancement of clinical decision making in HIV care in Africa | This is a review or systematic review |
| John Gregson | 2016 | Global epidemiology of drug resistance after failure of WHO recommended first-line regimens for adult HIV-1 infection: A multicentre retrospective cohort study | Patients not living in SSA |
| Erik Sörstedt | 2016 | Effect of dolutegravir in combination with Nucleoside Reverse Transcriptase Inhibitors (NRTIs) on people living with HIV who have pre-existing NRTI mutations | Patients not living in SSA |
| Orrell, Catherine | 2017 | Fixed-dose combination dolutegravir, abacavir, and lamivudine versus ritonavir-boosted atazanavir plus tenofovir disoproxil fumarate and emtricitabine in previously untreated women with HIV-1 infection (ARIA): week 48 results from a randomised, open-label, non-inferiority, phase 3b study | Patients not living in SSA |
| Dahourou Désiré Lucien | 2017 | Efavirenz-based simplification after successful early lopinavir-boosted-ritonavir-based therapy in HIV-infected children in Burkina Faso and Côte d'Ivoire: The MONOD ANRS 12206 non-inferiority randomised trial | Patients/participants are INSTI-unexperienced |
| Abram Michael E. | 2017 | Lack of impact of pre-existing T97A HIV-1 integrase mutation on integrase strand transfer inhibitor resistance and treatment outcome | Patients not living in SSA |
| Idris Abdullahi Nasir | 2017 | Human Immunodeficiency Virus Resistance Testing Technologies and Their Applicability in Resource-Limited Settings of Africa | This is a review or systematic review |
| Karolin Meixenberger | 2017 | Molecular evolution of HIV-1 integrase during the 20 years prior to the first approval of integrase inhibitors | Patients/participants are INSTI-unexperienced |
| George Mondinde Ikomey | 2017 | Observed HIV drug resistance associated mutations amongst naïve immunocompetent children in Yaoundé, Cameroon | Patients/participants are INSTI-unexperienced |
| Seth Inzaule | 2017 | Emergence of untreatable, multidrug-resistant HIV-1 in patients failing second-line therapy in Kenya | Patients/participants are INSTI-unexperienced |
| Elliot Raizes | 2017 | The US President’s Emergency Plan for AIDS Relief (PEPFAR) and HIV Drug Resistance: Mitigating Risk, Monitoring Impact | Title/Abstract irrelevant |
| Mohamed A. Daw | 2017 | Molecular and epidemiological characterization of HIV-1 subtypes among Libyan patients | Patients not living in SSA |
| Jennifer A. Fulcher | 2017 | Emergence of integrase resistance mutations during initial therapy with TDF/FTC/DTG. | Full text unclear or unavailable |
| Andrew N. Phillips | 2017 | Impact of HIV drug resistance on HIV/AIDS-associated mortality, new infections, and antiretroviral therapy program costs in Sub-Saharan Africa | Study did not involve participants |
| Michael R. Jordan | 2017 | HIV drug resistance in African infants and young children newly diagnosed with HIV: a multicounty analysis | Patients/participants are INSTI-unexperienced |
| Silvia Requena | 2017 | Drug resistance mutations in HIV-2 patients failing raltegravir and influence on dolutegravir response | Patients not living in SSA |
| Bethany Corrigan | 2017 | Characteristics of Treatment-Experienced HIV-infected African Children and Adolescents Initiating Darunavir and/or Etravirine-based Antiretroviral Treatment | Patients/participants are INSTI-unexperienced |
| Emmanuel Ndashimye | 2017 | High time to start human immunodeficiency virus type 1-infected patients on integrase inhibitors in sub-Saharan Africa | This is a review or systematic review |
| Aubin J. Nanfack | 2017 | Multimethod Longitudinal HIV Drug Resistance Analysis in Antiretroviral- Therapy-Naive Patients | Patients/participants are INSTI-unexperienced |
| Eva Natukunda | 2017 | Safety, efficacy, and pharmacokinetics of single-tablet elvitegravir, cobicistat, emtricitabine, and tenofovir alafenamide in virologically suppressed, HIV-infected children: a single-arm, open-label trial | Patients not living in SSA |
| Alexander Zoufaly | 2017 | Prevalence of integrase inhibitor resistance mutations in Austrian patients recently diagnosed with HIV from 2008 to 2013 | Patients not living in SSA |
| Catherine A Hankins | 2017 | Ending AIDS as a public health threat by 2030: Scientific Developments from the 2016 INTEREST Conference in Yaoundé, Cameroon | This is a review or systematic review |
| Juan Ambrosioni | 2017 | Integrase strand-transfer inhibitor polymorphic and accessory resistance substitutions in patients with acute/recent HIV infection | Patients not living in SSA |
| Ragna S. Boerma | 2017 | Multicentre analysis of second-line antiretroviral treatment in HIV-infected children : adolescents at high risk of failure | This is a review or systematic review |
| Ingeborg Wijting | 2017 | Dolutegravir as maintenance monotherapy for HIV (DOMONO): a phase 2, randomised non-inferiority trial | Patients not living in SSA |
| Luigia Elzi | 2017 | Adverse events of raltegravir and dolutegravir: A prospective analysis of 4,041 HIV-infected individuals from the Swiss HIV Cohort Study | Patients not living in SSA |
| Katherine J. Lepik | 2017 | Emergent drug resistance with integrase strand transfer inhibitor-based regimens | Patients not living in SSA |
| Pedro Cahn | 2017 | Candidates for inclusion in a universal antiretroviral regimen: Dolutegravir | This is a review or systematic review |
| Samantha Andreis | 2017 | Drug resistance in B and non-B subtypes amongst subjects recently diagnosed as primary/recent or chronic HIV-infected over the period 2013–2016: Impact on susceptibility to first-line strategies including integrase strand-transfer inhibitors | Patients not living in SSA |
| Yiannis Koullias | 2017 | Should We Be Testing for Baseline Integrase Resistance in Patients Newly Diagnosed With Human Immunodeficiency Virus? | Patients not living in SSA |
| Murat Sayan | 2017 | Integrase Strand Transfer Inhibitors (INSTIs) Resistance Mutations in HIV-1 Infected Turkish Patients | Patients not living in SSA |
| Willem D.F | 2017 | The ADVANCE study: a groundbreaking trial to evaluate a candidate universal antiretroviral regimen | This is a review or systematic review |
| Nachman, Sharon | 2018 | Safety and efficacy at 240 weeks of different raltegravir formulations in children with HIV-1: a phase 1/2 open label, non-randomised, multicentre trial | Patients not living in SSA |
| Anne Hoppe | 2018 | HIV-1 viral load and resistance in genital secretions in patients taking protease-inhibitor-based second-line therapy in Africa | Patients/participants are INSTI-unexperienced |
| Djenaba Fofana | 2018 | Resistance profile and treatment outcomes in HIV-infected children at virological failure in Benin, West Africa | Patients/participants are INSTI-unexperienced |
| Evans Denise | 2018 | Predictors of switch to and early outcomes on third-line antiretroviral therapy at a large public-sector clinic in Johannesburg, South Africa | Data Unclear (Authors weren't conntacted) |
| Vivian Black | 2018 | Issues about periconception use of dolutegravir are reminiscent of early concerns about efavirenz | This is a review or systematic review |
| Charlotte Charpentier | 2018 | Resistance to HIV integrase inhibitors: About R263K and E157Q mutations | This is a review or systematic review |
| Vitoria Marco | 2018 | The transition to dolutegravir and other new antiretrovirals in low-income and middle-income countries: What are the issues? | This is a review or systematic review |
| Nathan Osman | 2018 | Durable suppression of HIV-1 with resistance mutations to integrase inhibitors by dolutegravir following drug washout | Study did not involve participants |
| Vivian Black | 2018 | Issues about periconception use of dolutegravir are reminiscent of early concerns about efavirenz | This is a review or systematic review |
| Jienchi Dorward | 2018 | Dolutegravir for first-line antiretroviral therapy in low-income and middle-income countries: uncertainties and opportunities for implementation and research | This is a review or systematic review |
| Charlotte Charpentier | 2018 | High virological suppression regardless of the genotypic susceptibility score after switching to a dolutegravir-based regimen: Week 48 results in an observational cohort | Patients not living in SSA |
| Seth C. Inzaule | 2018 | Primary resistance to integrase strand transfer inhibitors in patients infected with diverse HIV-1 subtypes in sub-Saharan Africa | Patients/participants are INSTI-unexperienced |
| Seth C. Inzaule | 2018 | When prevention of mother-To-child HIV transmission fails: Preventing pretreatment drug resistance in African children | This is a review or systematic review |
| Alaoui Najwa | 2018 | Prevalence of resistance to integrase strand-transfer inhibitors (INSTIs) among untreated HIV-1 infected patients in Morocco | Patients not living in SSA |
| Marco Vitoria | 2018 | The transition to dolutegravir and other new antiretrovirals in low-income and middle-income countries: What are the issues? | This is a review or systematic review |
| Jean-Michel Molina | 2018 | Switching to fixed-dose bictegravir, emtricitabine, and tenofovir alafenamide from dolutegravir plus abacavir and lamivudine in virologically suppressed adults with HIV-1: 48 week results of a randomised, double-blind, multicentre, active-controlled, phase 3 non-inferiority trial | Patients not living in SSA |
| Fabien Fily | 2018 | HIV-1 drug resistance testing at second-line regimen failure in Arua, Uganda: avoiding unnecessary switch to an empiric third-line | Patients/participants are INSTI-unexperienced |
| Leonard Rogers | 2018 | Structural implications of genotypic variations in HIV-1 integrase from diverse subtypes | Study did not involve participants |
| Andrew N. Phillips | 2018 | Risks and benefits of dolutegravir-based antiretroviral drug regimens in sub-Saharan Africa: a modelling study | Study did not involve participants |
| Paula Vaz | 2018 | Compromise of Second-Line Antiretroviral Therapy Due to High Rates of Human Immunodeficiency Virus Drug Resistance in Mozambican Treatment-Experienced Children With Virologic Failure | Patients/participants are INSTI-unexperienced |
| Reneé de waal | 2018 | HIV drug resistance in sub-Saharan Africa: public health questions and the potential role of real-world data and mathematical modelling. | Study did not involve participants |
| James Nuttall | 2018 | Characteristics and early outcomes of children and adolescents treated with darunavir/ritonavir-, raltegravir- or etravirine-containing antiretroviral therapy in the Western Cape Province of South Africa | Full text unclear or unavailable |
| Joseph Fokam | 2018 | Next-generation sequencing provides an added value in determining drug resistance and viral tropism in Cameroonian HIV-1 vertically infected children | Patients/participants are INSTI-unexperienced |
| Eugenia Bruzzese | 2018 | Dolutegravir-based anti-retroviral therapy is effective and safe in HIV-infected paediatric patients | Patients not living in SSA |
| Jomy M. George | 2018 | Rapid Development of High-Level Resistance to Dolutegravir with Emergence of T97A Mutation in 2 Treatment-Experienced Individuals with Baseline Partial Sensitivity to Dolutegravir | Patients not living in SSA |
| Dominik Brado | 2018 | Analyses of HIV-1 integrase sequences prior to South African national HIV-Treatment program and available of integrase inhibitors in Cape Town, South Africa | Patients/participants are INSTI-unexperienced |
| Richard Kaplan | 2018 | Resistance to first-line ART and a role for dolutegravir | This is a review or systematic review |
| Maria Antonia De Francesco | 2018 | Prevalence of integrase strand transfer inhibitors (InSTIs) resistance mutations in InSTIs-naive and -experienced HIV-1 infected patients: a single Center experience | Patients not living in SSA |
| Perpetua Lum Tanyi | 2018 | Hiv/aids and older adults in cameroon: Emerging issues and implications for caregiving and policy-making | This is a review or systematic review |
| Kara S. McGee | 2018 | Canary in the Coal Mine? Transmitted Mutations Conferring Resistance to All Integrase Strand Transfer Inhibitors in a Treatment-Naive Patient | Patients/participants are INSTI-unexperienced |
| James Demarest | 2018 | Dolutegravir-Based Regimens Are Active in Integrase Strand Transfer Inhibitor–Naive Patients with Nucleoside Reverse Transcriptase Inhibitor Resistance | Patients not living in SSA |
| Gaëlle F. Tchouwa | 2018 | Nationwide Estimates of Viral Load Suppression and Acquired HIV Drug Resistance in Cameroon | Patients/participants are INSTI-unexperienced |
| Josep M Llibre | 2018 | Efficacy, safety, and tolerability of dolutegravir-rilpivirine for the maintenance of virological suppression in adults with HIV-1: phase 3, randomised, non-inferiority SWORD-1 and SWORD-2 studies | Patients not living in SSA |
| Serges Eholie | 2019 | Implementation of an intensive adherence intervention in patients with second-line antiretroviral therapy failure in four west African countries with little access to genotypic resistance testing: a prospective cohort study | Patients/participants are INSTI-unexperienced |
| Jennifer A. Thompson | 2019 | Evolution of protease inhibitor resistance in HIV-1-infected patients failing protease inhibitor monotherapy as second-line therapy in low-income countries: an observational analysis within the EARNEST randomised trial | Patients/participants are INSTI-unexperienced |
| Meriki Henry Dilonga | 2019 | Genetic diversity and antiretroviral resistance-associated mutation profile of treated and naive HIV-1 infected patients from the Northwest and Southwest regions of Cameroon | Patients/participants are INSTI-unexperienced |
| Rutstein Sarah E. | 2019 | High rates of transmitted NNRTI resistance among persons with acute HIV infection in Malawi: Implications for first-line dolutegravir scale-up | Patients/participants are INSTI-unexperienced |
| Yendewa George A. | 2019 | Prevalence of drug resistance mutations among ART-naive and-experienced HIV-infected patients in Sierra Leone | Patients/participants are INSTI-unexperienced |
| Smith Robert A. | 2019 | Comparison of the antiviral activity of bictegravir against HIV-1 and HIV-2 isolates and integrase inhibitor-resistant HIV-2 mutants | Study did not involve participants |
| Alaoui Najwa | 2019 | HIV-1 Integrase Resistance among Highly Antiretroviral Experienced Patients from Morocco | Patients not living in SSA |
| Havlir Diane V. | 2019 | Global HIV Treatment — Turning Headwinds to Tailwinds | This is a review or systematic review |
| Emmanuel Ndashimye | 2019 | The urgent need for more potent antiretroviral therapy in low-income countries to achieve UNAIDS 90-90-90 and complete eradication of AIDS by 2030 | This is a review or systematic review |
| Lucas de Almeida Machado | 2019 | Raltegravir-Induced Adaptations of the HIV-1 Integrase: Analysis of Structure, Variability, and Mutation Co-occurrence | Study did not involve participants |
| Komal Daipule | 2019 | Targeting Integrase Enzyme: A Therapeutic Approach to Combat HIV Resistance | This is a review or systematic review |
| Ume L. Abbas | 2019 | Drug resistance from preferred antiretroviral regimens for HIV infection in South Africa: A modeling study | Study did not involve participants |
| Stephen I. Walimbwa | 2019 | Drug Interactions between Dolutegravir and Artemether- Lumefantrine or Artesunate-Amodiaquine | Patients/participants are HIV-negative |
| Anne Derache | 2019 | Predicted antiviral activity of tenofovir versus abacavir in combination with a cytosine analogue and the integrase inhibitor dolutegravir in HIV-1-infected South African patients initiating or failing first-line ART | Patients/participants are INSTI-unexperienced |
| Salou Mounerou | 2019 | Challenges of scale-up to Dolutegravir based regimens in sub-saharan Africa: A case study in Togo | Patients/participants are INSTI-unexperienced |
| Ralph-Sydney Mboumba Bouassa | 2019 | High predictive efficacy of integrase strand transfer inhibitors in perinatally HIV-1-infected African children in therapeutic failure of first- A nd second-line antiretroviral drug regimens recommended by the WHO | Patients/participants are INSTI-unexperienced |
| Tennison Onoriode Digban | 2019 | Analyses of HIV-1 Integrase gene Sequences among treatment naive patients in the Eastern Cape, South Africa | Patients/participants are INSTI-unexperienced |
| Laurent Hocqueloux | 2019 | Dolutegravir Monotherapy Versus Dolutegravir/Abacavir/Lamivudine for Virologically Suppressed People Living With Chronic Human Immunodeficiency Virus Infection: The Randomized Noninferiority MONotherapy of TiviCAY Trial | Patients not living in SSA |
| Seth C. Inzaule | 2019 | Curbing the rise of HIV drug resistance in low-income and middle-income countries : the role of dolutegravir-containing regimens | This is a review or systematic review |
| Seth C. Inzaule | 2019 | Pretreatment HIV drug resistance in low- and middle-income countries | This is a review or systematic review |
| Lutresse Thome | 2019 | Échec Thérapeutique , Résistance Acquise du VIH et Souches Virales chez les Adultes sous Traitement Antirétroviral de Deuxième Ligne au Cameroun : Étude sur 18 ans ( 1999- 2017 ) de Monitorage à l ’ Hôpital Central de Yaoundé | Patients/participants are INSTI-unexperienced |
| Marta Alvarez | 2019 | Surveillance of transmitted drug resistance to integrase inhibitors in Spain: implications for clinical practice | Patients not living in SSA |
| Jienchi Dorward | 2019 | Dolutegravir in sub-Saharan Africa: context is crucial | This is a review or systematic review |
| Seth C. Inzaule | 2019 | Increasing levels of pretreatment HIV drug resistance and safety concerns for dolutegravir use in women of reproductive age | Patients not living in SSA |
| Vinie Kouamou | 2019 | Drug resistance and optimizing dolutegravir regimens for adolescents and young adults failing antiretroviral therapy | Patients/participants are INSTI-unexperienced |
| Anesu Marume | 2019 | Evaluation of the early warning indicators of HIV drug resistance surveillance system in Manicaland province, Zimbabwe | Patients/participants are HIV-negative |
| Landon Myer | 2019 | Periconception dolutegravir use in women living with HIV and missed opportunities in maternal and child health. | This is a review or systematic review |
| Segujja, Farouk | 2020 | High Levels of Acquired HIV Drug Resistance following Virological Nonsuppression in HIV-Infected Women from a High-Risk Cohort in Uganda | Patients/participants are INSTI-unexperienced |
| Moranguinho, Inês | 2020 | Genotypic resistance profiles of HIV-2-infected patients from Cape Verde failing first-line antiretroviral therapy | Patients/participants are INSTI-unexperienced |
| Brendan O’Kelly | 2020 | Therapeutic Drug Monitoring of HIV Antiretroviral Drugs in Pregnancy: A Narrative Review | This is a review or systematic review |
| Carmen de Mendoza | 2020 | Antiretroviral therapy for HIV-2 infection in non-endemic regions | Patients not living in SSA |
| Mohamed N'dongo Sangaré | 2020 | Impact of previous HIV resistance and virologic failures on virologic outcome following a switch to dolutegravir with 2 NRTIs among people living with HIV | Patients not living in SSA |
| Joseph Fokam | 2020 | Pre-treatment drug resistance and HIV-1 genetic diversity in the rural and urban settings of Northwest-Cameroon | Patients/participants are INSTI-unexperienced |
| Mabeya Sepha | 2020 | Characterization of HIV-1 Integrase Gene and Resistance Associated Mutations Prior to Roll out of Integrase Inhibitors by Kenyan National HIV-Treatment Program in Kenya | Patients/participants are INSTI-unexperienced |
| Boris K. Tchounga | 2020 | Survival among antiretroviral-experienced hiv-2 patients experiencing virologic failure with drug resistance mutations in Cote d'Ivoire West Africa | Patients/participants are INSTI-unexperienced |
| Aboubacar Soumah | 2020 | High rates of antiretroviral coverage and virological suppression in HIV-1-infected children and adolescents | Patients not living in SSA |
| Grant-McAuley Wendy | 2020 | Antiretroviral drug use and HIV drug resistance in female sex workers in Tanzania and the Dominican Republic | Patients/participants are INSTI-unexperienced |
| Strope Jonathan D. | 2020 | Drug-drug interactions in patients with hiv and cancer in sub-saharan Africa | Full text unclear or unavailable |
| Alhassan Yussif | 2020 | Community acceptability of dolutegravir-based HIV treatment in women: a qualitative study in South Africa and Uganda | Study did not involve participants |
| Mikasi Sello Given | 2020 | HIV-1 Integrase Diversity and Resistance-Associated Mutations and Polymorphisms among Integrase Strand Transfer Inhibitor-Naive HIV-1 Patients from Cameroon | Patients/participants are INSTI-unexperienced |
| Isaacs Darren | 2020 | Structural Comparison of Diverse HIV-1 Subtypes using Molecular Modelling and Docking Analyses of Integrase Inhibitors | Patients/participants are INSTI-unexperienced |
| Gabriela Patten | 2020 | Raltegravir use and outcomes among children and adolescents living with HIV in the IeDEA global consortium | Patients not living in SSA |
| João Esaú C. | 2020 | Raltegravir versus efavirenz in antiretroviral-naive pregnant women living with HIV (NICHD P1081): an open-label, randomised, controlled, phase 4 trial | Patients not living in SSA |
| Anthony Hauser | 2020 | Impact of scaling up dolutegravir on antiretroviral resistance in South Africa: A modeling study | Study did not involve participants |
| Marta Boffito | 2020 | Perspectives on the Barrier to Resistance for Dolutegravir + Lamivudine, a Two-Drug Antiretroviral Therapy for HIV-1 Infection | This is a review or systematic review |
| Mikasi Sello Given | 2020 | HIV-1 diversity and the implementation of integrase strand-transfer inhibitors as part of combination antiretroviral therapy | Study did not involve participants |
| Maria Lahuerta | 2020 | Monitoring the transition to new antiretroviral treatment regimens through an enhanced data system in Kenya | Study did not involve participants |
| Elizabeth D. Lowenthal | 2020 | Rapid initiation of dolutegravir for adults in Botswana | Full text unclear or unavailable |
| Janne Estill | 2020 | More evidence for dolutegravir as first-line ART for all | Study did not involve participants |
| Philip L. Tzou | 2020 | Integrase strand transfer inhibitor (INSTI)-resistance mutations for the surveillance of transmitted HIV-1 drug resistance | Study did not involve participants |
| Mabeya Sepha | 2020 | Characterization of HIV-1 Integrase Gene and Resistance Associated Mutations Prior to Roll out of Integrase Inhibitors by Kenyan National HIV-Treatment Program in Kenya | Patients/participants are INSTI-unexperienced |
| Obasa Adetayo Emmanuel | 2020 | Drug Resistance Mutations Against Protease, Reverse Transcriptase and Integrase Inhibitors in People Living With HIV-1 Receiving Boosted Protease Inhibitors in South Africa | Patients/participants are INSTI-unexperienced |
| Kate El Bouzidi | 2020 | High prevalence of integrase mutation L74I in West African HIV-1 subtypes prior to integrase inhibitor treatment. | Patients/participants are INSTI-unexperienced |
| Pablo Ferrer | 2020 | HIV-1 resistance patterns to integrase inhibitors in Chilean patients with virological failure on raltegravir-containing regimens | Patients not living in SSA |
| Aurelio Orta‑Resendiz | 2020 | HIV-1 acquired drug resistance to integrase inhibitors in a cohort of antiretroviral therapy multi-experienced Mexican patients failing to raltegravir: A cross-sectional study | Patients not living in SSA |
| Andrew N. Phillips | 2020 | Updated assessment of risks and benefits of dolutegravir versus efavirenz in new antiretroviral treatment initiators in sub-Saharan Africa: modelling to inform treatment guidelines | This is a review or systematic review |
| Salim Masoud | 2020 | Circulating HIV-1 Integrase Genotypes in Tanzania: Implication on the Introduction of Integrase Inhibitors-Based Antiretroviral Therapy Regimen | Patients/participants are INSTI-unexperienced |
| Yussif Alhassan | 2020 | Engendering health systems in response to national rollout of dolutegravir-based regimens among women of childbearing potential: a qualitative study with stakeholders in South Africa and Uganda | Study did not involve participants |
| Ezechiel Semengue | 2021 | Baseline integrase drug resistance mutations ans conserved regions accross HIV 1 clades in Cameroon: implications for transition to dolutegravir in ressource y limited settings | Patients/participants are INSTI-unexperienced |
| Sello Given Mikasi | 2021 | HIV-1 Drug Resistance Mutation Analyses of Cameroon-Derived Integrase Sequences | Patients/participants are INSTI-unexperienced |
| Benjamin M. Wenk | 2021 | Prevalence of integrase strand transfer inhibitor resistance mutations in antiretroviral-naive HIV-1-infected individuals in Cameroon | Patients/participants are INSTI-unexperienced |
| Kim Steegen | 2021 | Is there a role for doravirine in African HIV treatment programmes? A large observational resistance study in South Africa | Full text unclear or unavailable |
| Marina Rubio-Garrido | 2021 | High drug resistance levels could compromise the control of HIV infection in paediatric and adolescent population in Kinshasa, the Democratic Republic of Congo | Patients/participants are INSTI-unexperienced |
| Moore Cecilia L. | 2021 | ODYSSEY clinical trial design: a randomised global study to evaluate the efficacy and safety of dolutegravir-based antiretroviral therapy in HIV-positive children, with nested pharmacokinetic sub-studies to evaluate pragmatic WHO-weight-band based dolutegravir dosing | Patients not living in SSA |
| Mikasi Sello Given | 2021 | Interaction analysis of statistically enriched mutations identified in Cameroon recombinant subtype CRF02_AG that can influence the development of Dolutegravir drug resistance mutations. | Patients/participants are INSTI-unexperienced |
| Herieth Ismael Wilson | 2021 | Rollout of dolutegravir-based antiretroviral therapy in sub-Saharan Africa and its public health implications | Study did not involve participants |
| Juliana da Silva | 2021 | Monitoring emerging HIV drug resistance in sub-Saharan Africa in the era of dolutegravir | This is a review or systematic review |
| Ying Zhao | 2021 | AntiRetroviral Therapy In Second-line: investigating Tenofovir-lamivudine-dolutegravir (ARTIST): protocol for a randomised controlled trial | Paper is a study protocol |
