# Supplementary material for: HIV-1 integrase resistance associated mutations and the use of dolutegravir in Sub-Saharan Africa: A systematic review and meta-analysis
Source: PLOS Glob Public Health. 2022 Oct 11;2(10):e0000826. doi: 10.1371/journal.pgph.0000826 (PMC10021461; doi:10.1371/journal.pgph.0000826)
Supplement: Attachment — Submitted filename: Response to reviewers.docx [file pgph.0000826.s003.docx]

**RESPONSE TO REVIEWERS**

Journal Requirements:

1. Please provide separate figure files in .tif or .eps format only and remove any figures embedded in your manuscript file. Please ensure that all files are under our size limit of 20MB. For more information about how to convert your figure files please see our guidelines: Once you've converted your files to .tif or .eps, please also make sure that your figures meet our format requirements:

<https://journals.plos.org/globalpublichealth/s/figures>

**Response**: *We are very appreciative for this comment and have converted all our figures in .tif files.*

2. Please update the 'Competing Interests' statement in the system with "The authors have declared that no competing interests exist".

**Response**: *We updated the statement in the system as suggested.*

3. Please amend your detailed Financial Disclosure statement. This is published with the article. It must therefore be completed in full sentences and contain the exact wording you wish to be published.

a. Please clarify all sources of funding (financial or material support) for your study. List the grants (with grant number) or organizations (with url) that supported your study, including funding received from your institution.

**Response**: *We thank the reviewer for this comment*. *Our funding source is clearly stated within the manuscript after the acknowledgment section (See line 354).*

b. State the initials, alongside each funding source, of each author to receive each grant.

**Response**: *Not applicable. Our home institution (CIRCB) supported all cost-related to the research.*

c. State what role the funders took in the study. If the funders had no role in your study, please state: “The funders had no role in study design, data collection and analysis, decision to publish, or preparation of the manuscript.”

**Response**: *We thank the reviewers for this suggestion. We added the sentence as suggested (see lines 363-365).*

d. If any authors received a salary from any of your funders, please state which authors and which funders. If you did not receive any funding for this study, please simply state: “The authors received no specific funding for this work.”

**Response**: *We thank the reviewers for this suggestion. We added the sentence as suggested (see lines 363-365).*

4. Figure 7: please (a) provide a direct link to the base layer of the map used and ensure this is also included in the figure legend; (b) provide a link to the terms of use / license information for the base layer. We cannot publish proprietary or copyrighted maps (e.g. Google Maps, Mapquest) and the terms of use for your map base layer must be compatible with our CC-BY 4.0 license. If your map was obtained from a copyrighted source please amend the figure so that the base map used is from an openly available source. Alternatively, please provide explicit written permission from the copyright holder granting you the right to publish the material under our CC-BY 4.0 license. Please note that the following CC BY licenses are compatible with PLOS license: CC BY 4.0, CC BY 2.0 and CC BY 3.0, meanwhile such licenses as CC BY-ND 3.0 and others are not compatible due to additional restrictions. If you are unsure whether you can use a map or not, please do reach out and we will be able to help you. The following websites are good examples of where you can source open access or public domain maps:

* U.S. Geological Survey (USGS) - All maps are in the public domain. (<http://www.usgs.gov>)

* PlaniGlobe - All maps are published under a Creative Commons license so please cite “PlaniGlobe, <http://www.planiglobe.com>, CC BY 2.0” in the image credit after the caption. (<http://www.planiglobe.com/?lang=enl>)

* Natural Earth - All maps are public domain. (<http://www.naturalearthdata.com/about/terms-of-use/>)

**Response**: *We thank the reviewers for these suggestions. We amended the figure and inserted the link of the base layer as well as a written permission as suggested.*

5. In the online submission form, you indicated that "The data that support the findings of this study are available from the corresponding author, upon reasonable request.". All PLOS journals now require all data underlying the findings described in their manuscript to be freely available to other researchers, either 1. In a public repository, 2. Within the manuscript itself, or 3. Uploaded as supplementary information. This policy applies to all data except where public deposition would breach compliance with the protocol approved by your research ethics board. If your data cannot be made publicly available for ethical or legal reasons (e.g., public availability would compromise patient privacy), please explain your reasons by return email and your exemption request will be escalated to the editor for approval. Your exemption request will be handled independently and will not hold up the peer review process, but will need to be resolved should your manuscript be accepted for publication. One of the Editorial team will then be in touch if there are any issues.

**Response**: *We thank the reviewers for this comment. The sentence is indeed misleading and we removed it in the system and in the manuscript (see lines 355-356).*

6. We have noticed that you have uploaded Supporting Information files, but you have not included a list of legends. Please add a full list of legends for your Supporting Information files after the references list.

**Response**: *We thank the reviewers for this suggestion. We added a list of legends as suggested (See lines 552-559).*

Please review your reference list to ensure that it is complete and correct. If you have cited papers that have been retracted, please include the rationale for doing so in the manuscript text, or remove these references and replace them with relevant current references. Any changes to the reference list should be mentioned in the rebuttal letter that accompanies your revised manuscript. If you need to cite a retracted article, indicate the article’s retracted status in the References list and also include a citation and full reference for the retraction notice.

Additional Editor Comments (if provided):

[Note: HTML markup is below. Please do not edit.]

Reviewers' comments:

Reviewer's Responses to Questions

Comments to the Author

1. Does this manuscript meet PLOS Global Public Health’s publication criteria? Is the manuscript technically sound, and do the data support the conclusions? The manuscript must describe methodologically and ethically rigorous research with conclusions that are appropriately drawn based on the data presented.

Reviewer #1: Yes

Reviewer #2: Yes

2. Has the statistical analysis been performed appropriately and rigorously?

Reviewer #1: Yes

Reviewer #2: Yes

3. Have the authors made all data underlying the findings in their manuscript fully available (please refer to the Data Availability Statement at the start of the manuscript PDF file)?

The PLOS Data policy requires authors to make all data underlying the findings described in their manuscript fully available without restriction, with rare exception. The data should be provided as part of the manuscript or its supporting information, or deposited to a public repository. For example, in addition to summary statistics, the data points behind means, medians and variance measures should be available. If there are restrictions on publicly sharing data—e.g. participant privacy or use of data from a third party—those must be specified.

Reviewer #1: Yes

Reviewer #2: Yes

4. Is the manuscript presented in an intelligible fashion and written in standard English?

PLOS Global Public Health does not copyedit accepted manuscripts, so the language in submitted articles must be clear, correct, and unambiguous. Any typographical or grammatical errors should be corrected at revision, so please note any specific errors here.

Reviewer #1: Yes

Reviewer #2: Yes

5. Review Comments to the Author

Please use the space provided to explain your answers to the questions above. You may also include additional comments for the author, including concerns about dual publication, research ethics, or publication ethics. (Please upload your review as an attachment if it exceeds 20,000 characters)

Reviewer #1: The paper is well written, clearly organized and the topic and the findings are important. My only suggestion is there are a lot of abbreviations and that they do not enhance readability, quite the contrary.

**Response**: *We thank the reviewer for this comment. The manuscript have been revised throughout and we reduced the use of abbreviations.*

Reviewer #2: Thank you for writing this paper. I have searched for the equivalent a number of times and find myself trying to keep tally on the papers out there. It is a relief and very appreciated that you have done the work and written this review. It's very important. Appreciate the discussion of subtypes and genetic diversity.

To add to the paper, would consider adding some of these points into discussion/intro. They do not need an indepth dive but are all factors that impact this paper and its implications:

1. Could you add more on prevalence of HIV relative to resistance? The total number with HIV and the prevalence are much higher in some regions than others. I think showing this weighting will help those who are working from a policy angle alone. Even just a mention would help.

**Response**: *We thank the reviewer for this comment. We added a comment on HIVDR prevalence in the discussion section (see lines 267-269 and references 53-55).*

2. Could you add more on roll out level? This is obviously a dynamic process, but it will be interesting to see how the resistance levels change as roll out is further. Can you also discuss the amount of raltegravir use? How much EVG has been used in Senegal? Clearly other integrase inhibitors are not common choices so interesting if can give a sense of the amount of use to have an idea of the impact.

**Response**: *We thank the reviewer for this comment. However, the purpose of this paper was to discuss on the use of DTG before the transition in SSA and thus we could go further to see the effect of DTG-roll out on resistance levels. Also, the use of RAL and EVG have been discussed according to data available before the transition.*

3. Could you discuss more on HIV-2? I was glad to see this raised. There has always been the question of how EFV based regimens impacted HIV-2 resistance, given the risk of misdiagnosis as HIV-1 in some early testing algorithms and missed dual infection, though not high prevalence still an interesting question.

**Response**: *We thank the reviewer for this comment. We totally agree with the reviewer as many authors reported non-effectiveness of EFV (or other NNRTI) in the presence of HIV-2 infection. However, within the scope of this systematic review, we analyzed and discussed only data available regarding the use of INSTI; and questions related to HIV-2 in that domain have being accordingly addressed.*

4. Discussion, any further thoughts on the progression of this. recommendations? model predictions? dynamic process, where see going? thoughts on models, key observations for what they should include to ensure accurate consideration of baseline and dynamics

https://journals.plos.org/plosmedicine/article?id=10.1371/journal.pmed.1003397

**Response**: *We thank the reviewer for this comment. The aforementioned study was excluded from the review (see appendix 2) because it is a modelling study and thus it served only for predictions and did not actually include patients in the design. Furthermore, prediction models were certainly considered while framing WHO recommendations.*

5. anything on dolutegravir and adherence, interruptions, robustness to adherence?

**Response**: *We thank the reviewer for this comment. We first did not addressed issues on adherence under DTG-based in SSA as studies included in the review did not report data on adherence. However we acknowledged this point as a limitation in the current version of the manuscript and added a comment accordingly (see lines 332-336 and reference 71).*

6. Would bring up other meds? ie as given as TLD. this means resistance to TDF is a concern. baseline resistance to other meds? mono therapy risk? any thought not this. without detail, may be worth mentioining

<https://pubmed.ncbi.nlm.nih.gov/31895149/>

**Response**: *We thank the reviewer for this comment. We completely agree with the reviewer about the concern regarding DTG functional mono-therapy due to accumulation of NRTI-resistance mutations in patients transitioning to TLD. However, updated data inside and out of SSA reported a good virological response of DTG-based even in the advent of pre-existing resistance to NRTI (or especially TDF). Results from cohort studies addressing this are still awaited to bring forth evidence and clear all reasonable doubts.*

7. Other potential papers

https://journals.lww.com/aidsonline/Fulltext/2021/12152/Adherence,_resistance,_and_viral_suppression_on.4.aspx Adherence, resistance, and viral suppression on dolutegravir in sub-Saharan Africa: implications for the TLD era

Adherence, resistance, and viral suppression on dolutegravir in sub-Saharan Africa: implications for the TLD era Dolutegravir drug-resistance monitoring in Africa (if have access)

<https://journals.plos.org/plosmedicine/article?id=10.1371/journal.pmed.1003397>; <https://pubmed.ncbi.nlm.nih.gov/31895149/>;

**Response**: *We thank the reviewer for these suggestions. We added one reference as suggested (see reference 71).*

Table 1, Figure 5 and 6. why are first and last names used to note studies? **Response**: *We thank the reviewer for this comment. First and last names were used to note studies because we did not have any other way to present the result nor did we have any recommendations for that.*

Figure 5 - could another term be used instead of prevalence for achievement of virology control. It could be misread. frequency, virologic control etc

**Response**: *We thank the reviewer for this comment. The figure has been revised accordingly.*

Figure 7 - would clarify for readers, it makes it look like all of HIV in West Africa is HIV-2. Appreciate HIV-2 and resistance being brought up but would also want to make sure prevalence/distribution clear to readers

**Response**: *We thank the reviewer for this comment. The figure has been revised accordingly. Moreover, we added a comment regarding this point in the main text (see lines 309-312).*

6. PLOS authors have the option to publish the peer review history of their article (what does this mean?). If published, this will include your full peer review and any attached files.

Do you want your identity to be public for this peer review? If you choose “no”, your identity will remain anonymous but your review may still be made public.

For information about this choice, including consent withdrawal, please see our Privacy Policy.

Reviewer #1: No

Reviewer #2: No

Once again, we really appreciate all the reviewers’ inputs, as we are convinced that they have really improved the quality of our manuscript.
